# Fevers

**Published:** 1905-04-29

**Authors:** 


### Progress in Medicine and Surgery.

### FEVERS.

(*Continued from page* 54.)

**Typhoid Fever.**—The attention of some members of the profession on the other side of the Atlantic is being directed to the question of pure water-supply as being a highly important factor in the prevention of typhoid fever.[21] Many instances can be adduced in Great Britain, Germany, and elsewhere of towns in which the typhoid incidence has been greatly lessened by the effective filtration of water. J. D. Craig shows also that the same good effect has followed the adoption of the sand filtration of water in some cities in the United States. In Chicago the typhoid rate fell from 159 to 46 per 100,000 population after providing a less contaminated water-supply, and in Albany the number of reported cases fell from an average of 489 per annum in the four years preceding filtration to one of 109 in the four years succeeding its introduction. The municipalities of many American cities exhibit a curious and amazing apathy on the subject. In Philadelphia [22] a wealthy and prosperous city, with fine wide streets, and freedom from poverty and overcrowding, the death-rate, which should be low, is over 19·13 per 1,000 of the population. In 1900 the population was set down at 1,293,700, and in 1903 the reported cases of typhoid numbered 8,701 with 957 deaths. Sometimes as many as 300 cases of typhoid fever are notified in a week. The only drinking-water available is that of the Delaware and Schuylkill rivers which is delivered unfiltered to the inhabitants. The latter is a small river which receives above the Philadelphia intake the crude sewage of several manufacturing towns, including that of Reading, which numbers 79,000 inhabitants. Philadelphia is an ardent water-drinking community, and ice-water and ice-creams are much in vogue, hence the need of a purer water-supply is pressing. Filtration beds are now in process of construction. A similar state of things exists in Washington, the political capital of a great nation, a city of spacious gardens and wide avenues without poverty or unhealthy industry, yet where the death-rate is 21·51 per 1,000. Filter beds are at last in course of construction. In Pittsburg,[23] the "iron city," and in Allegheny, on the opposite side of the Allegheny River, cities dominated by the iron kings and oil lords, a similar or worse state of affairs exists. Altogether, the sewage of 500,000 to 600,000 persons drains directly into the Pittsburg water-supply without any attempt at purification. Until 1898 the Allegheny intake was below that of Pittsburg. "A terrible sewer, known as 'butcher's run sewer,' because it received the offal from many slaughter-houses, then emptied into the river above the Allegheny water intake. When the annual clearing of the reservoirs was made, dead cats, dogs, and chickens, large pieces of flesh and other organic matter in decomposition were pumped out." The great prevalence of typhoid fever led to the construction of a main higher up the river, which has somewhat improved matters. Pittsburg water-still receives the hospital drain and that of an asylum for the aged less than two miles above its point of intake!

Mr. C. Rucker [24] draws attention to the fact that Schottmüller discovered the bacillus of Eberth in the blood in 84 per cent. of 119 cases, frequently before the Widal test was positive, and in one case as early as the second day. Where available this is, therefore, an early and unimpeachable test for enteric fever. Initial infection may take place through the tonsils, as Eberth's bacillus has been repeatedly isolated from these organs, and tonsillitis and laryngitis occasionally occur early in the disease. Ehrlich's diazo-reaction, which may be first obtained from the fifth to the thirteenth day, is chiefly valuable as a negative sign, since it may occur also in measles, scarlet fever, variola, pneumonia, septic conditions, and malignant disease. It also occurs as the result of the administration of certain drugs as shown by Campanella [25] and others. Iodides, bark, and tannin may prohibit the reaction, while salol, codeine, resorcin, creosote, quinine, digitalis, chrysarobin and naphthalin may produce it. The action may also be greatly intensified by the excessive presence of urochrome, indican, and diacetic acid in the urine. G. Gubb [26] reports a case of a febrile enteric fever, followed by thrombosis of the left femoral vein. The Widal reaction was positive. Recent writers have ascribed the polyuria,[27] noticed long ago by Murchison to be of very frequent occurrence in convalescence from typhoid fever, to nervous origin. The chief feature in the tropics is, according to Biddle,[28] a marked hæmorrhagic tendency. It is stated that an abundant typhoid rash, forming the "exanthematic" type of the disease, is of favourable import, the intensity of the exanthem and of the intestinal symptoms being in inverse proportion.

The treatment of enteric formed the subject of the last Bradshaw lecture which was delivered by Dr. Foord Caiger,[29] who confined his remarks entirely to the field of therapeutics. Treatment is dealt with under the three headings of specific, antipyretic, and antiseptic. The complications of hæmorrhage and perforation also receive notice. Attempts to secure an antitoxin similar to the diphtheria and tetanus antitoxins by inoculation of the horse have hitherto failed. Such experiments have produced a serum possessing antibacterial but not antitoxic properties. Chantemesse, however, claims to have produced a serum possessing real curative powers. This has been employed by himself and by several other French observers. In a total of 696 cases so treated the mortality was only 5 per cent. The nature of this serum is not quite clear. Chantemesse,[30] who has himself treated 545 cases, with only 22 deaths (4 per cent.), says that the serum produces an energetic specific action on the spleen, lymphoid tissue, and bone marrow—the defensive apparatus of the organism—which is more vigorous the earlier it is produced. Its action is peculiar in that the more profoundly the patient is affected by the disease the weaker, not the stronger, must be the dose. As Caiger points out, the evidence of the value of the serum is weakened by the fact that other measures, such as packs and baths, were employed simultaneously. Caiger is hopeful that Wright's prophylactic vaccine inoculations will lead to good results, and that the initial period of increased susceptibility, carefully pointed out by Wright himself, will be shortened by improved methods. Caiger believes that, in limits, the fever of typhoid is the natural defence of the organism against bacterial invasion, and that as such it is beneficial. Quinine is the only antipyretic drug he favours at all. The bath treatment is highly spoken of, not only for its antipyretic, but also for its tonic, and especially its eliminative effect. The acknowledged increased liability to relapse and the inconvenience of the treatment are the chief drawbacks. The latter objection applies especially to private practice, where also the disinclination of the patient and the prejudices of his friends form formidable difficulties which the physician should, however, seek to overcome in cases which really demand the treatment. Tepid baths, graduated baths, wetpacks and cold effusions have all their good points and their advocates, but cannot seriously rival the cold bath as a systematic method of treatment. The lecturer considers that it is undeniable that the antiseptic method, though frequently misunderstood, is founded on a scientific basis. During an experience of 15 years he has tried most of the intestinal antiseptics in vogue and has usually been disappointed. Of these sulphurous acid in doses of 20 to 30 minims every two or three hours, oil of turpentine, and the combination of quinine and nascent chlorine recommended by Burney Yeo are regarded with most favour. The essential oil of cinnamon recommended by J. C. Ross of Withington has also so far given good results, fever and intestinal decomposition are reduced, and the drug has a marked soothing soporific effect. Two or three minims of cinnamon oil distilled from the bark should be given every two hours in emulsion, or in gelatine capsule. The purely expectant attitude of *laissez faire* Caiger condemns as bad treatment. For diarrhœa an enema of half a drachm of laudanum in two ounces of thin starch gruel is preferred, and a large cold compress, frequently repeated, to the abdomen is very beneficial. Watch should be kept for meteorism, a signal of great danger. The diet must be revised. Ice to the abdomen and moderate doses of opium may prove successful, or if the colon is at fault a soap and water enema with turpentine. The pulse must be watched with great care. As a rule, alcohol is considered harmful, except when certain conditions arise. For hæmorrhage absolute rest, a full dose of opium, an ice-bag to the abdomen, and the *complete deprivation of fluids*, except for an occasional fragment of ice, are advised. R. M. Harbin [31] advocates therapeutic fasting. After 24 or 48 hours fast broths, cocoa, egg albumin, coffee, etc., are given in definite quantities. Milk must only be given diluted with from two to five parts of cocoa, coffee, broth, water, etc. The mortality based on 116 cases was only 3·4 per cent. in Harbin's hands.

[21] Med. Record, Oct. 1, p. 538. [22] Lancet, Nov. 5, p. 1315. [23] Ibid., Nov. 19, p. 1451. [24] Med. Record, Nov. 12, p. 779. [25] Ibid., Oct. 29, p. 700. [26] Brit. Med. Jour., Dec. 3, p. 1511. [27] Lancet, Oct. 22, p. 1167. [28] Med. Record, Nov. 19, p. 821. [29] Lancet, Nov. 26. [30] Med. Record, Nov. 12, p. 782. [31] Ibid., p. 771.

(*To be continued.*)

